# MicroRNA-520d-3p suppresses melanoma cells proliferation by inhibiting the anti-silencing function 1B histone chaperone

**DOI:** 10.1080/21655979.2021.2001914

**Published:** 2021-12-07

**Authors:** Xian Shi, Xidan Xu, Nian Shi, Yongjun Chen, Manni Fu

**Affiliations:** Department of Dermatology, Huangshi Central Hospital, Huangshi, China

**Keywords:** MiR-520d-3p, *ASF1B*, melanoma, proliferation, apoptosis, adhesion

## Abstract

As the most common and aggressive malignant form of skin cancer, melanoma has a poor prognosis in its late stage. MicroRNA (miR)-520d-3p has been reported as a key modulator that regulates the development of different types of cancer, but its role in melanoma remains unclear. The purpose of this study was to investigate the role and mechanism of miR-520d-3p in melanoma. The expression of anti-silencing function 1B histone chaperone (*ASF1B*) and miR-520d-3p in melanoma tissues and cells was detected by reverse transcription-quantitative polymerase chain reaction. The interaction between *ASF1B* and miR-520d-3p was verified by luciferase activity detection. Cell counting kit-8, bromodeoxyuridine, fluorescein isothiocyanate, and cell adhesion assays were performed to detect cell viability, proliferation, apoptosis, and adhesion in melanoma cells. *ASF1B* expression was evidently increased, whereas miR-520d-3p level was downregulated in melanoma tissues and cells. Overexpression of *ASF1B* enhanced cell growth and adhesion and hampered cell apoptosis in melanoma cells. Furthermore, miR-520d-3p suppressed the tumorigenic effects of melanoma cells. Moreover, miR-520d-3p suppressed the expression of *ASF1B* to suppress melanoma tumorigenesis. In conclusion, we have found out that miR-520d-3p suppressed melanoma tumorigenesis by inhibiting *ASF1B*, which could be a promising target for melanoma therapy.

## Introduction

Melanoma is the most common and aggressive malignancy of skin cancer, with high incidence, metastatic rate, and mortality worldwide [1,2]. Although early stage melanoma is partly curable by surgical resection, advanced melanoma has poor prognosis [3]. Therefore, accurate diagnosis in the early stage is essential for improving the prognosis of melanoma.

The anti-silencing function 1B histone chaperone (*ASF1B*) gene is located on chromosome 19q13.12 and consists of four exons. It encodes a member of the H3/H4 family of histone chaperone proteins, which may play a key role in modulating the nucleosome structure of chromatin by constantly supplying histones at sites of nucleosome assembly [4,5]. *ASF1B* has been reported to be implicated in various cellular functions related to cancer pathogenesis and progression, such as cervical cancer, prostate cancer, and clear cell renal cell carcinoma, which could enhance cell growth and migration, but reduce cell apoptosis [4,6,7]. However, the role of *ASF1B* in melanoma has not been clearly elucidated.

MicroRNAs (miRNAs), approximately 22 nucleotides in length, are an abundant class of small non-coding RNAs that serve as gene regulators by preventing mRNA translation or mRNA degradation [8]. Different miRNAs functioning as tumor suppressors or oncogenes contribute to human cancer cell growth and apoptosis [9,10]. The miR-520d-3p has been reported as a key modulator in different types of cancers by regulating cell growth, migration, invasion, and apoptosis, such as breast cancer, hepatocellular carcinoma, gastric cancer, and ovarian cancer [11–14]. Nevertheless, whether miR-520d-3p plays a role in the progression of melanoma remains unclear.

In this study, we have attempted to obtain a thorough understanding of the mechanism of the miR-520d-3p-*ASF1B* axis in melanoma. We studied the effect of the miR-520d-3p-*ASF1B* axis on the malignant behavior of melanoma cells *in vitro* and found that miR-520d-3p suppresses *ASF1B* to hamper the progression of melanoma based on the targeted regulatory mechanisms of miRNAs and mRNAs. Our study may deepen our understanding of the miR-520d-3p-*ASF1B* axis in melanoma development and highlights the potential of the miR-520d-3p-*ASF1B* axis as a new target for melanoma therapy.

## Materials and methods

### Tissue samples, cell lines, and cell transfection

A total of 38 melanoma and normal tissues from patients with melanoma were collected from our hospital with informed consent and approved by our hospital’s ethics committee. The inclusion criteria for our study included patients diagnosed with melanoma by pathology and without a history of melanoma. The exclusion criteria were organ dysfunction, other malignant tumors, other skin diseases, and infectious and autoimmune diseases.

The human normal skin cell line HaCAT and melanoma cell lines (A375, WM35, A875, and A2058) were purchased from ATCC (Manassas, VA, USA) and maintained in Dulbecco’s modified Eagle’s medium (DMEM; Gibco, USA) containing 10% fetal bovine serum (FBS; Gibco, USA) at 37°C with 5% CO_2_.

SiRNA-*ASF1B* (50 nM), overexpression *ASF1B* (2 μg/m), miR-520d-3p inhibitor (75 nM), mimic (50 nM), and negative control were purchased from GenePharma (Shanghai, China), and the A375 and A875 cells were transfected with the transfections using Lipo2000 (Invitrogen, USA). After 48 h, other functional studies were performed.

### RNA extraction and reverse transcription-quantitative polymerase chain reaction

The total mRNA of the tissues and cells was extracted using the Total RNA Extraction Kit (A27828, Thermo, USA). cDNA was transcribed using the Maxima First Strand cDNA Synthesis Kit (K1642, Thermo, USA). Subsequently, the Applied Biosystems PowerUp SYBR Green (Thermo, USA) was assessed by quantitative reverse transcription-polymerase chain reaction (RT-qPCR). The miRNA of the tissues and cells was extracted using the miRcute miRNA extraction kit (DP501, Tiangen, China). The cDNA was transcribed using the miRcute miRNA First Strand cDNA Synthesis Kit (KR211, Tiangen, China). Subsequently, RT-qPCR was performed using the miRcute Enhanced miRNA Fluorescence Quantitative Detection Kit (FP411, Tiangen, China). Relative *ASF1B* expression was normalized to glyceraldehyde-3-phosphate dehydrogenase (GAPDH), and miR-520d-3p expression was normalized to small RNA U6 (U6). The data were analyzed using the 2-^ΔΔCt^ method [15]. All primer sequences are listed in Table 1.

**Table 1.** The sequence of PCR primers used in this study

Primer name	Sequence
ASF1B	Forward:5ʹ-GATCAGCTTCGAGTGCAGTG-3ʹ
	Reverse:5ʹ-TGGTAGGTGCAGGTGATGAG-3ʹ
miR-193b-3p	Forward:5ʹ-GCGCAACTGGCCCTCAAAGT-3ʹ
	Reverse:5ʹ-GTGCAGGGTCCGAGGT-3ʹ
GAPDH	Forward:5ʹ-CCATCTTCCAGGAGCGAGAT-3’
	Reverse:5ʹ-TGCTGATGATCTTGAGGCTG-3’
U6	Forward:5ʹ-CGCTTCACGAATTTGCGTGTCAT-3’
	Reverse:5ʹ-TATGGAACGCTTCACGAATTTG-3’

### Western blotting analysis

Cells were lysed using radioimmunoprecipitation assay lysis buffer (Beyotime, China) and quantified using a bicinchoninic acid (BCA) kit (Cat#: #23235, Thermo, USA). Thirty micrograms of protein from each sample was loaded on 10% sodium dodecyl sulfate polyacrylamide gel electrophoresis and electroblotted onto a polyvinylidene fluoride (PVDF) membrane. Anti-*ASF1B* (Cat#: ab183651, Abcam, UK) and anti-GAPDH (Cat#: ab181602, Abcam, UK) were diluted in Tris-buffered saline with 0.1% (v/v) Tween 20 (TBST) at 1:1,000, and the membranes were incubated with the antibodies overnight at 4°C. After washing, the membranes were incubated with anti-HRP rabbit antibody diluted in TBST at 1:10,000. Band intensity was detected using an electrochemiluminescent system (Bio-Rad, USA) and Gel-Pro Analyzer 4.0 (Media Cybernetics, USA) [16].

### Cell counting kit-8 assay

Cells of 5 × 10^3^ density were cultured in 96-well plates. At four time points, that is, 0, 24, 48, and 72 h, and the medium was replaced with 90 µL fresh medium, and 10 µL CCK-8 working solution (Cat#: ab228554; Abcam, UK) was added to each well and incubated for another 2 h. The OD value was examined using a multimode plate reader (Thermo, USA) at each time point at a wavelength of 450 nm [7].

### Bromodeoxyuridine assay

A375 and A875 cells of 2 × 10^4^ density were cultured in 96-well plates with serum-free medium overnight. The next day, cells were maintained for 24 h in medium containing 10% FBS. Then, the BrdU Cell Proliferation Assay Kit (Cat#: 6813, CST, MA, USA) was used to detect cell proliferation. BrdU (10 μM) was added to the plate and incubated for 4 h and then incubated with secondary antibody for another 1 h. The OD value was examined using a multimode plate reader at a wavelength of 450 nm [17].

### Apoptosis assay

As described in a study [18], fluorescein isothiocyanate (FITC) Annexin V Apoptosis Detection Kit was used to detect apoptosis in A375 and A875 cells (Cat#: 556547; BD, USA). Approximately 6 × 10^4^ cells were harvested and suspended in 100 µL binding buffer; 5 µL FITC and 5 µL propidium iodide were added to the buffer and maintained in the dark for 15 min. After washing twice, cells were suspended in the binding buffer, detected by flow cytometry, and analyzed using FlowJo (Tree Star, USA).

### Caspase 3 activity assay

A375 and A875 cells (2 × 10^4^) were cultured in 96-well plates. A caspase-3 activity assay kit (Cat#: 5723, CST, USA) was used to detect cell apoptosis. Cells were collected in the cell lysis buffer, and caspase-3 assay loading solution was added for 2 h at 37°C. Subsequently, the OD value was detected on a multimode-plate reader at a wavelength of 405 nm [19].

### Cell adhesion assay

Cell adhesion assay was performed based on a previous study [20]. Collagen I solution (Cat#: C7661, Sigma, USA) was used to prepare the 96-well cell adhesion plate. A375 and A875 cells (2 × 10^4^) were cultured in 96-well plates. The next day, cells were washed and maintained in serum-free DMEM for 8 h. Subsequently, the cells were treated with 10 mM ethylenediaminetetraacetic acid (EDTA) for 10 min to dissociate the cells and suspended in DMEM (2 × 10^5^ cells/mL). Then, the cell suspension was added to the cell adhesion plate and incubated for 20 min at 37°C. After the cells adhered to the surface, non-adherent cells were washed away and incubated with DMEM containing 10% FBS for 4 h at 37°C. Subsequently, 10 μL of 3-(4, 5-dimethylthiazol-2-yl)-2, 5-diphenyl tetrazolium bromide substrate (Cat#: C0009S, Beyotime, China) was added to the cells and incubated for 2 h. Then, 100 µL dimethyl sulfoxide was added to the cells, and the OD value was detected on a multimode-plate-reader (Thermo, USA) at a wavelength of 570 nm.

### Luciferase assay

We purchased the pmiRGLO vectors with wild-type or mutated *ASF1B* 3′-untranslated regions (UTRs) from Tuoran Bio (Shanghai, China). A375 and A875 cells were cultured in a 24-well plate (2 × 10^5^ cells); furthermore, we transfected the one of the vectors and either miR-NC or miR-520d-3p into the cells with Lipo2000. Cells were harvested and measured using the Luciferase Assay Kit (Cat#: RG027, Beyotime, China) after 72 h. Renilla activity was used as an internal control [21].

### Statistical analysis

The experiment was performed thrice and the data are summarized as mean ± standard deviation. Independent sample *t*-tests were evaluated for two-group comparisons and one-way analysis of variance for multiple comparisons with GraphPad 6.0 (GraphPad, USA). The association between *ASF1B* and miR-520d-3p expression was evaluated using Pearson correlation analysis. Statistical significance was set at *P* < 0.05.

## Results

In this study, we hypothesized that *ASF1B* is tumorigenic in melanoma and is targeted by miR-520d-3p. We analyzed *ASF1B* levels in melanoma tissues and cell lines. In addition, to investigate the role of *ASF1B* in melanoma, we examined the effects of *ASF1B* knockdown on the proliferation, apoptosis, and adhesion of melanoma cells. Moreover, we verified the targeted binding between *ASF1B* and miR-520d-3p, and further explored the role of *ASF1B* in the survival of melanoma cells mediated by miR-520d-3p.

### The identification of downstream ASF1B of miR-520d-3p

**Figure 1.**
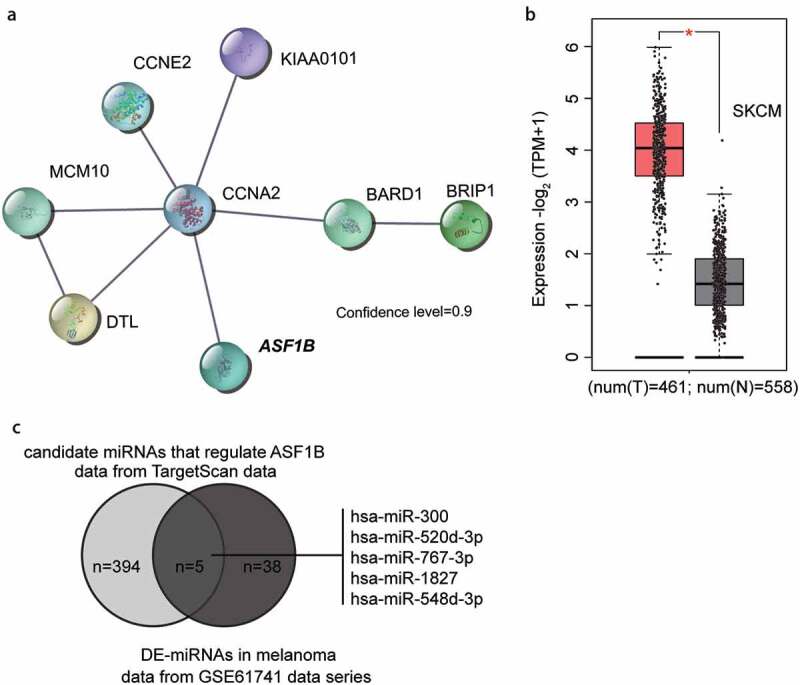
The identification of ASF1B as the downstream effector of miR-520d-3p. (a) The protein-protein interaction network of the top 30 significantly downregulated genes of GSE18512 data series. Eight genes were shown in the network. (b) The expression of ASF1B in melanoma (data from GEPIA database). T: tumor; N: normal. (c) The intersection between the target miRNAs of ASF1B predicted by targetscan algorithm and differentially expressed miRNAs (DE-miRNAs) in melanoma from GSE61741 microarray analysis using the criteria of adjusted P < 0.05 and logFC≤-1.5. X

miR-193b has been reported to be involved in melanoma progression [22–24]. To further explore the downstream of miR-193b involving the key mRNAs, GSE18512 from GEO DataSets (https://www.ncbi.nlm.nih.gov/geo/) was selected because it stored the mRNA expression data in the melanoma cell samples with miR-193b inhibitor and melanoma cell samples with inhibitor negative control. By analyzing the GSE18512 data series, we identified 78 significantly downregulated genes after miR-193-3p upregulation. We selected the top 30 most downregulated genes (Table 2) and uploaded them to the STRING database (https://string-db.org/). Protein–protein interaction network analysis showed that eight genes (*CCNA2, CCNE2, ASF1B, KIAA0101, MCM10, DTL, BARD1*, and *BRIP1*) were closely correlated with strong confidence (Figure 1(a)). Among the eight genes, we found that *ASF1B* was significantly upregulated in melanoma (Figure 1(b), data from GEPIA database, http://gepia2.cancer-pku.cn/). In addition, *ASF1B* has been suggested to be a significant promoter of tumor in other cancers [4,6,7] but has not been studied in melanoma. In addition, miR-193b could be identified as an miRNA that targets *ASF1B* by GSE18512. Therefore, to further identify the candidate miRNAs regulating *ASF1B*, we intersected the miRNAs predicted by TargetScan algorithm (version: 7.2) and the differentially expressed miRNAs in melanoma from GSE61741 with the criteria of adjusted *P* < 0.05, and logFC ≤ −1.5. Five common miRNAs were identified: hsa-miR-300, hsa-miR-520d-3p, hsa-miR-767-3p, hsa-miR-1827, and hsa-miR-548d-3p (Figure 1(c)). Among the five miRNAs, miR-520d-3p attracted our attention because of its anticancer effect in multiple cancers [11,13,14] and for the reason that no study has been performed to find out its effect in melanoma [25].

**Table 2.** The top 30 significantly downregulated genes from GSE18515 data series using the criteria of P < 0.05 and logFC≤-1.5

Probe ID	P value	logFC	Gene symbol	Gene title
A_32_P124245	0.000204	−2.82132055	OLIG3	oligodendrocyte transcription factor 3
A_24_P62833	0.043112	−2.2809665	ADAMTSL4	ADAMTS like 4
A_24_P332314	0.001149	−2.27329825	FAM111B	family with sequence similarity 111 member B
A_23_P200866	0.000129	−2.1609278	STMN1	stathmin 1
A_24_P345002	0.00059	−2.13749766	NUDT11	nudix hydrolase 11
A_23_P215976	0.001305	−2.1352401	CCNE2	cyclin E2
A_23_P334414	0.000235	−2.05563853	TRAF3IP3	TRAF3 interacting protein 3
A_23_P163079	0.002018	−2.03622415	GCH1	GTP cyclohydrolase 1
A_23_P10385	0.000328	−2.02051663	DTL	denticleless E3 ubiquitin protein ligase homolog
A_23_P255328	0.001409	−1.9933815	PRDM8	PR/SET domain 8
A_32_P167904	0.000316	−1.93607401	ZNF681	zinc finger protein 681
A_23_P32165	0.000381	−1.87170315	LHX2	LIM homeobox 2
A_23_P32029	0.000475	−1.87117505	SLC35D2	solute carrier family 35 member D2
A_23_P202390	0.005488	−1.86520408	SUV39H2	suppressor of variegation 3–9 homolog 2
A_23_P119254	0.000275	−1.8571117	ASF1B	anti-silencing function 1B histone chaperone
A_24_P196351	0.001241	−1.84510351	PLXNC1	plexin C1
A_23_P158239	0.000459	−1.84255217	SHMT2	serine hydroxymethyltransferase 2
A_23_P115091	0.0004	−1.84135267	RAB25	RAB25, member RAS oncogene family
A_24_P247044	0.037349	−1.84102246	ZNF492	zinc finger protein 492
A_24_P280029	0.000383	−1.83075425	PDXP	pyridoxal phosphatase
A_32_P181222	0.002861	−1.81815147	KCNMA1	potassium calcium-activated channel subfamily M alpha 1
A_24_P932757	0.003572	−1.81636928	KIAA0101	KIAA0101
A_23_P394259	0.000319	−1.80758645	MPP2	membrane palmitoylated protein 2
A_23_P12816	0.003771	−1.7971523	HELLS	helicase, lymphoid-specific
A_23_P140316	0.005347	−1.79603862	GPR137C	G protein-coupled receptor 137 C
A_23_P58321	0.001263	−1.77915314	CCNA2	cyclin A2
A_23_P215070	0.000314	−1.7781434	CEP41	centrosomal protein 41
A_23_P67771	0.001378	−1.75346587	BARD1	BRCA1 associated RING domain 1
A_23_P15844	0.011843	−1.7482505	BRIP1	BRCA1 interacting protein C-terminal helicase 1
A_24_P412088	0.00046	−1.74457908	MCM10	minichromosome maintenance 10 replication initiation factor

### The expression of ASF1B was elevated in melanoma

**Figure 2.**
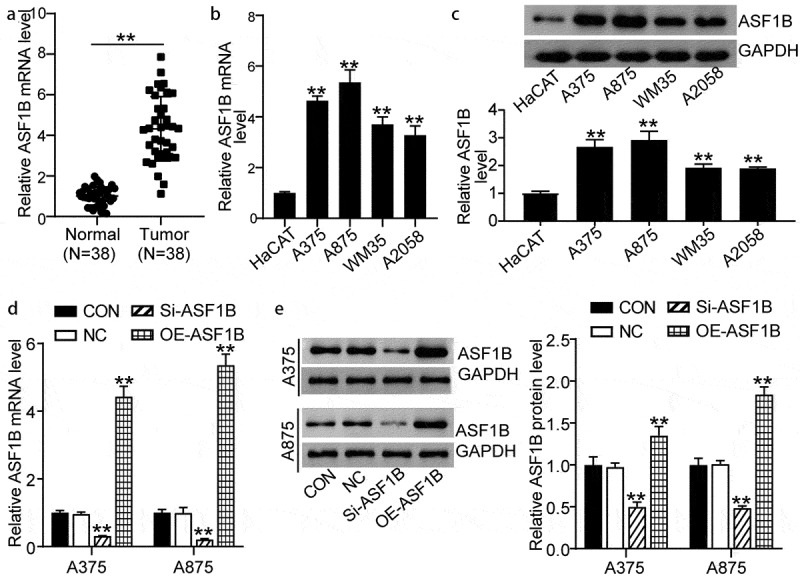
Upregulation of ASF1B in melanoma. (a) RT-qPCR detection of mRNA expression of ASF1B in melanoma tissues (N = 38) and normal tissues (N = 38). (b) RT-qPCR detection of mRNA expression of ASF1B in normal skin cell line (HaCAT) and melanoma cell lines (A375, WM35, A875, and A2058). (c) Measurement of ASF1B protein level in HaCAT, A375, WM35, A875, and A2058 cell lines by western blotting assay. (d) RT-qPCR detection of mRNA expression of ASF1B in A375 and A875 cells transfected with NC, Si-ASF1B and OE-ASF1B. (e) Western blotting detection of protein expression of ASF1B in A375 and A875 cells transfected with NC, Si-ASF1B and OE-ASF1B. *, *P* < 0.05; **, *P* < 0.001. CON, blank control; NC, negative control; Si-ASF1B, SiRNA-ASF1B; OE-ASF1B, overexpression-ASF1B

To assess the effect of *ASF1B* in melanoma, we first detected the expression of *ASF1B* in 38 pairs of tumor and normal tissues from patients with melanoma. *ASF1B* expression in the melanoma tissues was obviously 4-fold higher than that in normal tissues (1.00 ± 0.44 in normal tissues, 4.32 ± 1.59 in tumor tissues) (Figure 2(a)). Meanwhile, the expression of *ASF1B* in melanoma cells (A875, A375, WM35, and A2058) was dramatically higher than that in normal HaCAT cells; in particular, A375 and A875 cells showed the highest levels of these cell lines (1.00 ± 0.05 in HaCAT, 4.64 ± 0.18 in A375, 5.36 ± 0.49 in A875, 3.28 ± 0.37 in WM35, and 4.32 ± 1.59 in A2058 cells) (Figure 2(b)). Similarly, the melanoma cells also had significantly increased *ASF1B* protein levels compared to normal HaCAT cells (1.00 ± 0.08 in HaCAT, 2.68 ± 0.26 in A375, 2.92 ± 0.32 in A875, 1.92 ± 0.13 in WM35, and 0.89 ± 0.06 in A2058) (Figure 2(c)). Thus, we selected the A375 and A875 cell lines for further analysis. We transfected Si-NC, Si-*ASF1B*, and OE-*ASF1B* into the A375 and A875 cells. The OE-*ASF1B* cells exhibited by 5-fold upregulated *ASF1B* gene and by 1.5-fold increased protein expression, while the Si-*ASF1B* cells displayed over 50% downregulated *ASF1B* gene and protein expression relative to controls (Figure 2(d-e)) (Figure 2(d): 1.00 ± 0.07 in CON, 0.97 ± 0.05 in NC, 0.32 ± 0.01 in Si-*ASF1B*, 4.44 ± 0.30 in OE-*ASF1B* of A375 cells; 1.00 ± 0.10 in CON, 1.00 ± 0.15 in NC, 0.21 ± 0.02 in Si-*ASF1B*, 5.37 ± 0.32 in OE-*ASF1B* of A875 cells. Figure 2(e): 1.00 ± 0.09 in CON, 0.98 ± 0.04 in NC, 0.50 ± 0.06 in Si-*ASF1B*, 1.35 ± 0.11 in OE-*ASF1B* of A375 cells; 1.00 ± 0.08 in CON, 1.01 ± 0.04 in NC, 0.49 ± 0.02 in Si-*ASF1B*, 1.75 ± 0.15 in OE-*ASF1B* of A875 cells).

### ASF1B facilitated the development of melanoma in vitro

**Figure 3.**
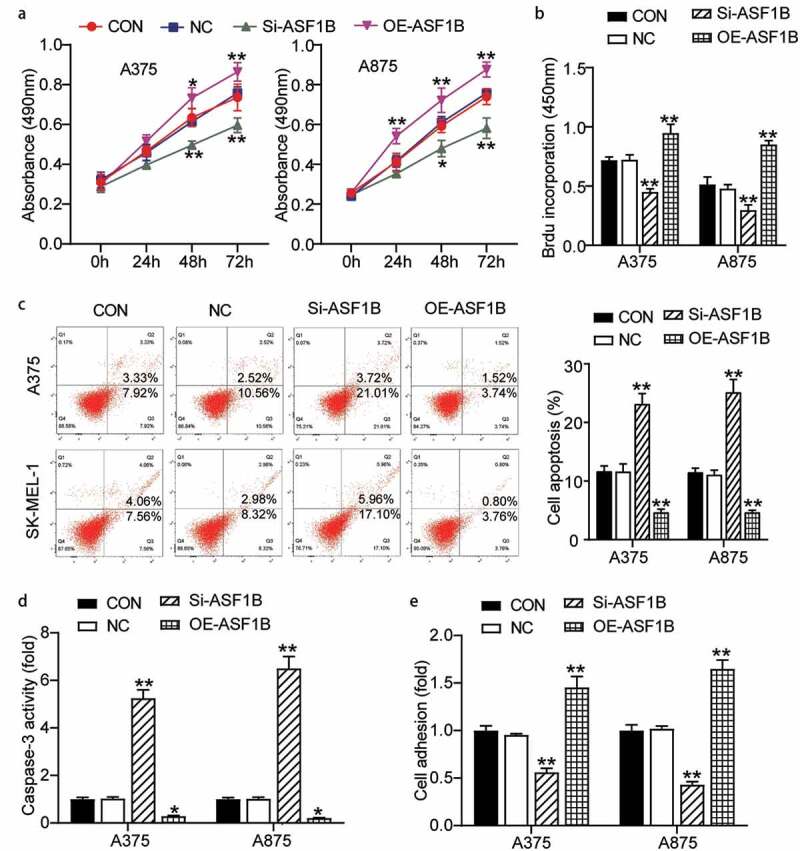
ASF1B facilitated proliferation and adhesion, but repressed cell apoptosis of melanoma cells. (a) Cell viability was detected in A375 and A875 cells transfected with NC, Si-ASF1B and OE-ASF1B by CCK8 assay. (b) Cell proliferation was detected in A375 and A875 cells transfected with NC, Si-ASF1B and OE-ASF1B by BrdU assay. (c) Cell apoptosis was determined in A375 and A875 cells transfected with NC, Si-ASF1B and OE-ASF1B by FITC apoptosis detection kit. (d) Cell apoptosis was determined in A375 and A875 cells transfected with NC, Si-ASF1B and OE-ASF1B by caspase-3 activity assay kit. (e) Cell adhesion was detected in A375 and A875 cells transfected with NC, Si-ASF1B and OE-ASF1B by cell adhesion assay kit. *, *P* < 0.05; **, *P* < 0.001. CON, blank control; NC, negative control; Si-ASF1B, SiRNA-ASF1B; OE-ASF1B, overexpression-ASF1B

We further validated the function of *ASF1B* in melanoma, the OE-*ASF1B* groups exhibited obviously higher cell viability, while the Si-*ASF1B* groups presented significant lower cell viability than control cells (0.74 ± 0.07 in CON, 0.76 ± 0.03 in NC, 0.60 ± 0.04 in Si-*ASF1B*, 0.86 ± 0.05 in OE-*ASF1B* of A375 cells at 72 h; 0.74 ± 0.04 in CON, 0.76 ± 0.02 in NC, 0.58 ± 0.05 in Si-*ASF1B*, 0.88 ± 0.04 in OE-*ASF1B* of A875 cells at 72 h) (Figure 3(a)). At the same time, two-fold increased cell proliferation in the OE-*ASF1B* groups, while 30% reduced cell proliferation in the Si-*ASF1B* groups relative to controls (0.72 ± 0.03 in CON, 0.72 ± 0.04 in NC, 0.45 ± 0.03 in Si-*ASF1B*, 0.95 ± 0.08 in OE-*ASF1B* of A375 cells; 0.51 ± 0.06 in CON, 0.48 ± 0.03 in NC, 0.30 ± 0.04 in Si-*ASF1B*, 0.85 ± 0.03 in OE-*ASF1B* of A875 cells) (Figure 3(b)). Subsequently, cell apoptosis level in the Si-*ASF1B* groups was elevated remarkably by 2-fold, but the OE-*ASF1B* groups suppressed 50% cell apoptosis compared with control cells by FITC assay and the same tendency was found by caspase 3 activity assay detection (Figure 3(c,d)) (Figure 3(c): 11.70 ± 0.89 in CON, 11.65 ± 1.29 in NC, 23.19 ± 1.74 in Si-*ASF1B*, 4.68 ± 0.53 in OE-*ASF1B* of A375 cells; 11.54 ± 0.68 in CON, 11.11 ± 0.75 in NC, 25.19 ± 2.15 in Si-*ASF1B*, 4.65 ± 0.33 in OE-*ASF1B* of A875 cells. Figure 3(d): 1.00 ± 0.07 in CON, 1.03 ± 0.06 in NC, 5.25 ± 0.36 in Si-*ASF1B*, 0.29 ± 0.03 in OE-*ASF1B* of A375 cells; 1.00 ± 0.07 in CON, 1.02 ± 0.07 in NC, 6.51 ± 0.50 in Si-*ASF1B*, 0.21 ± 0.02 in OE-*ASF1B* of A875 cells). Additionally, the OE-*ASF1B* groups exhibited dramatically increased cell adhesion, in contrast, the Si-*ASF1B* groups had significant downregulated cell adhesion relative to controls (1.00 ± 0.05 in CON, 0.96 ± 0.01 in NC, 0.56 ± 0.04 in Si-*ASF1B*, 1.45 ± 0.11 in OE-*ASF1B* of A375 cells; 1.00 ± 0.06 in CON, 1.02 ± 0.03 in NC, 0.43 ± 0.03 in Si-*ASF1B*, 1.65 ± 0.09 in OE-*ASF1B* of A875 cells) (Figure 3(e)). Hence, *ASF1B* facilitated cell growth and hampered cell apoptosis in melanoma.

### MiR-520d-3p targeted to ASF1B in melanoma

**Figure 4.**
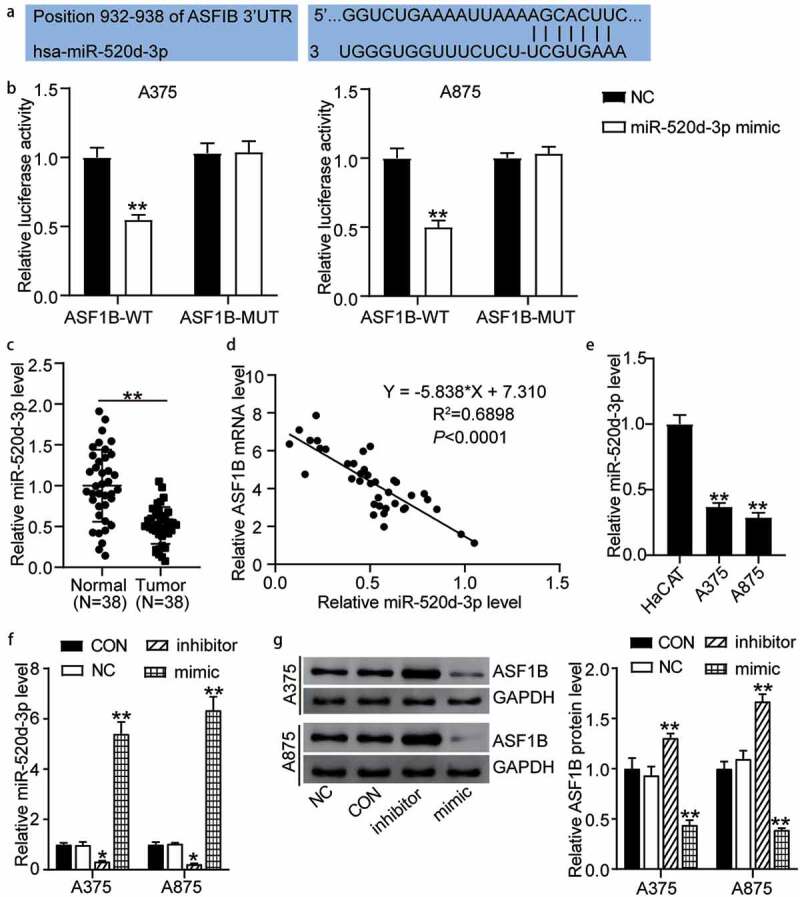
MiR-520d-3p targeting ASF1B inhibited the expression of ASF1B in melanoma. (a) TargetScan analysis showed the predicted binding site of ASF1B 3ʹ-UTR for miR-520d-3p. (b) Dual luciferase assay was performed in pmiRGLO ASF1B-WT and pmiRGLO ASF1B-MUT with treatment of NC or miR-520d-3p mimic. (c) RT-qPCR detection of expression of miR-520d-3p in melanoma tissues and (N = 38) normal tissues (N = 38). (d) Pearson’s correlation analysis of ASF1B mRNA levels and miR-520d-3p in melanoma tissues. (e) RT-qPCR detection of expression of miR-520d-3p in HaCAT, A375 and A875 cells. (f) RT-qPCR detection of expression of miR-520d-3p in A375 and A875 cells transfected with NC, miR-520d-3p inhibitor and miR-520d-3p mimic. (g) Measurement of ASF1B protein level in A375 and A875 cells transfected with NC, miR-520d-3p inhibitor and miR-520d-3p mimic. *, *P* < 0.05; **, *P* < 0.001. ASF1B-WT, ASF1B-wild-type; ASF1B-MUT, ASF1B-mutunt; CON, blank control; NC, negative control; inhibitor, miR-520d-3p inhibitor; mimic, miR-520d-3p mimic

The miR-520d-3p matching on position 932–938 of *ASF1B* 3ʹ-UTRs was predicted by TargetScan (Figure 4(a)). Furthermore, the detection of luciferase activity confirmed that miR-mimic in the pmiRGLO *ASF1B*-WT cells resulted in 50% decreased activity compared to cells transfected with miR-NC (1.00 ± 0.07 in NC, 0.55 ± 0.03 in miR-520d-3p mimic of A375 cells treated with *ASF1B*-WT; 1.00 ± 0.07 in NC, 0.50 ± 0.05 in miR-520d-3p mimic of A875 cells treated with *ASF1B*-WT). However, miR-520d-3p had no effect on the pmiRGLO *ASF1B*-MUT cells (1.03 ± 0.07 in NC, 1.04 ± 0.08 in miR-520d-3p mimic of A375 cells treated with *ASF1B*-MUT; 1.00 ± 0.03 in NC, 1.03 ± 0.05 in miR-520d-3p mimic of A875 cells treated with *ASF1B*-MUT) (Figure 4(b)). Additionally, the miR-520d-3p level in 38 melanoma tissues showed approximately 50% decreased levels compared with healthy controls (1.00 ± 0.44 in normal tissues, 0.51 ± 0.23 in tumor tissues) (Figure 4(c)). Furthermore, *ASF1B* expression was negatively correlated with miR-520d-3p expression in melanoma tissues (Figure 4(d)). The A375 and A875 melanoma cells presented over 60% miR-520d-3p levels than HaCAT cells (1.00 ± 0.07 in HaCAT, 0.37 ± 0.03 in A375, 0.29 ± 0.04 in A875) (Figure 4(e)). We further transfected miR-520d-3p NC, inhibitor, and mimic into A375 and A875 cells. The mimic groups displayed about 6-fold increased miR-520d-3p levels, while the inhibitor groups showed 80% downregulated levels relative to controls (1.00 ± 0.06 in CON, 0.98 ± 0.03 in NC, 0.33 ± 0.03 in inhibitor, 5.41 ± 0.47 in mimic of A375 cells; 1.00 ± 0.10 in CON, 1.03 ± 0.04 in NC, 0.22 ± 0.03 in inhibitor, and 6.35 ± 0.53 in mimic of A875 cells) (Figure 4(f)). Likewise, the mimic groups had 50% decreased *ASF1B* protein levels, while the inhibitor groups presented approximately 1.5-fold levels compared to control cells (1.00 ± 0.11 in CON, 0.97 ± 0.05 in NC, 1.31 ± 0.04 in inhibitor, 0.44 ± 0.05 in mimic of A375 cells; 1.00 ± 0.07 in CON, 1.06 ± 0.04 in NC, 1.67 ± 0.07 in inhibitor, 0.39 ± 0.02 in mimic of A875 cells) (Figure 4(g)). These results indicate that miR-520d-3p targeting *ASF1B* inhibits the expression of *ASF1B* in melanoma.

### MiR-520d-3p hampered melanoma cell progression by inhibiting ASF1B

**Figure 5.**
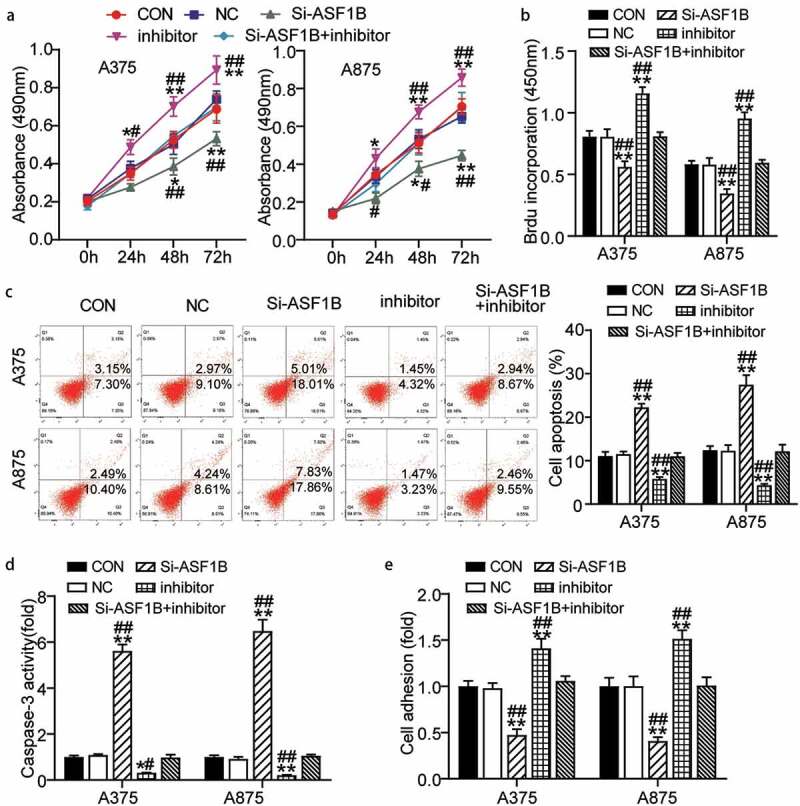
MiR-520d-3p hampered melanoma cell progression by inhibiting ASF1B. (a) Cell viability was detected in A375 and A875 cells transfected with NC, Si-ASF1B, miR-520d-3p inhibitor and Si-ASF1B+miR-520d-3p inhibitor by CCK8 assay. (b) Cell proliferation was detected in A375 and A875 cells transfected with NC, Si-ASF1B, miR-520d-3p inhibitor and Si-ASF1B+miR-520d-3p inhibitor by BrdU assay. (c) Cell apoptosis was determined in A375 and A875 cells transfected with NC, Si-ASF1B, miR-520d-3p inhibitor and Si-ASF1B+miR-520d-3p inhibitor by FITC apoptosis detection kit. (d) Cell apoptosis was determined in A375 and A875 cells transfected with NC, Si-ASF1B, miR-520d-3p inhibitor and Si-ASF1B+miR-520d-3p inhibitor by caspase-3 activity assay kit. (e) Cell adhesion was detected in A375 and A875 cells transfected with NC, Si-ASF1B, miR-520d-3p inhibitor and Si-ASF1B+miR-520d-3p inhibitor by cell adhesion assay kit. *, *P* < 0.05; **, *P* < 0.001. CON, blank control; NC, negative control; Si-ASF1B, SiRNA-ASF1B; inhibitor, miR-520d-3p inhibitor

Furthermore we examined whether the miR-520d-3p-*ASF1B* axis plays a role in melanoma. First, the inhibitor groups showed higher cell viability than control cells since 48 h, while this effect was weakened by the Si-*ASF1B* treatment (0.69 ± 0.07 in CON, 0.74 ± 0.04 in NC, 0.53 ± 0.04 in Si-*ASF1B*, 0.89 ± 0.07 in inhibitor, 0.69 ± 0.07 in Si-*ASF1B*+ inhibitor of A375 cells at 72 h; 0.70 ± 0.04 in CON, 0.65 ± 0.03 in NC, 0.45 ± 0.03 in Si-*ASF1B*, 0.86 ± 0.05 in inhibitor, 0.70 ± 0.08 in Si-*ASF1B*+ inhibitor of A875 cells at 72 h) (Figure 5(a)). Meanwhile, the inhibitor groups presented 1.5-fold increased cell proliferation compared to control cells, while this effect was weakened by the Si-*ASF1B* treatment (0.81 ± 0.05 in CON, 0.81 ± 0.06 in NC, 0.56 ± 0.04 in Si-*ASF1B*, 1.16 ± 0.05 in inhibitor, 0.81 ± 0.04 in Si-*ASF1B*+ inhibitor of A375 cells; 0.58 ± 0.03 in CON, 0.58 ± 0.05 in NC, 0.35 ± 0.04 in Si-*ASF1B*, 0.95 ± 0.05 in inhibitor, 0.59 ± 0.02 in Si-*ASF1B*+ inhibitor of A875 cells) (Figure 5(b)). Furthermore, the inhibitor groups showed 50% and 80% reduced apoptosis level compared to control cells, while this effect was weakened by the Si-*ASF1B* treatment by FITC assay and caspase-3 activity assay detection, respectively (Figure 5(c,d)) (Figure 5(c): 11.09 ± 0.92 in CON, 11.51 ± 0.06 in NC, 22.27 ± 0.84 in Si-*ASF1B*, 5.82 ± 0.39 in inhibitor, 11.02 ± 0.77 in Si-*ASF1B*+ inhibitor of A375 cells; 12.42 ± 0.91 in CON, 12.29 ± 1.29 in NC, 27.48 ± 2.20 in Si-*ASF1B*, 4.35 ± 0.31 in inhibitor, 12.16 ± 1.52 in Si-*ASF1B*+ inhibitor of A875 cells. Figure 5(d): 1.00 ± 0.06 in CON, 1.09 ± 0.04 in NC, 5.62 ± 0.28 in Si-*ASF1B*, 0.31 ± 0.01 in inhibitor, 0.98 ± 0.12 in Si-*ASF1B*+ inhibitor of A375 cells; 1.00 ± 0.07 in CON, 0.93 ± 0.09 in NC, 6.48 ± 0.49 in Si-*ASF1B*, 0.21 ± 0.02 in inhibitor, 1.06 ± 0.05 in Si-*ASF1B*+ inhibitor of A875 cells). Moreover, the inhibitor groups disclosed 1.5-fold elevated cell adhesion compared to control cells, while this effect was weakened by the Si-*ASF1B* treatment (1.00 ± 0.05 in CON, 0.98 ± 0.05 in NC, 0.47 ± 0.06 in Si-*ASF1B*, 1.41 ± 0.11 in inhibitor, 1.06 ± 0.06 in Si-*ASF1B*+ inhibitor of A375 cells; 1.00 ± 0.09 in CON, 1.00 ± 0.11 in NC, 0.41 ± 0.04 in Si-*ASF1B*, 1.52 ± 0.09 in inhibitor, 1.01 ± 0.09 in Si-*ASF1B*+ inhibitor of A875 cells) (Figure 5(e)). Overall, miR-520d-3p hampered melanoma cell progression by inhibiting *ASF1B*.

## Discussion

Although early stage melanoma is partly curable by surgical resection, advanced melanoma has poor prognosis [26]. Recognizing the importance of melanoma, researchers have focused on improving the effectiveness of melanoma treatment through early and accurate screening and diagnosis [27]. In this study, we have revealed the mechanism of the miR-520d-3p-*ASF1B* axis in melanoma, providing a valuable theoretical basis for early screening and diagnosis of melanoma. In this study, we have shown that *ASF1B* expression was increased, while miR-520d-3p expression was reduced in melanoma tissues and cells. Upregulation of *ASF1B* enhanced cellular proliferation and adhesion, but triggered a decline in cellular apoptosis. Furthermore, miR-520d-3p upregulation reduced proliferation and adhesion, but elevated apoptosis in melanoma cells. Moreover, miR-520d-3p inhibited *ASF1B* to suppress melanogenesis.

Recent findings have greatly expanded our understanding of the role of *ASF1B* in the pathogenesis of various cancers, including prostate cancer, clear cell renal cell carcinoma, and cervical cancer [4,6,7]. Recent studies indicated that *ASF1B* was overexpressed in clear cell renal cell carcinoma, and its upregulation boosted cancer cell growth by activating the AKT pathway [4,28]. Han et al. revealed that knockdown of *ASF1B* induced prostate cancer cell apoptosis by repressing the PI3K/Akt pathway [6]. Liu et al. suggested that the overexpression of *ASF1B* facilitated cervical cancer development by stabilizing CDK9 [7]. In the current study, we elucidated the role of *ASF1B* in melanoma progression. We found an obvious increase in *ASF1B* expression in melanoma tissues and cells and induced *ASF1B* knockdown and overexpression in melanoma cells. The results suggested that *ASF1B* effectively elevated cellular proliferation and adhesion and hampered cellular apoptosis in melanoma cells.

Numerous attempts have been made to uncover the role of miRNAs in melanoma development by regulating cell growth and apoptosis. Evidence has shown that miR-942-5p accelerates human melanoma cell growth by reducing DKK3 expression [29]. Qiu et al. clarified that miRNA-138 downregulates hypoxia-inducible factor 1α and hampers melanoma cell growth and metastasis [30]. Furthermore, knockdown of miR-126-3p contributed to dabrafenib-acquired resistance in melanoma by elevating ADAM9 and VEGF-A [31]. Several studies have demonstrated the pivotal role of miR-520d-3p in cancer initiation and development, such as in breast cancer, hepatocellular carcinoma, and gastric cancer [11,13,14]. For instance, knockdown of miR-520d-3p promoted cell growth by inhibiting long non-coding RNA MIAT and EPHA2 expression in hepatocellular carcinoma [14]. In addition, Ren et al. found that miR-520d-3p prevented post-transcriptional regulation of spindle and kinetochore-associated 2 expression in breast cancer [13]. Furthermore, miRNA 520d-3p inhibits cell growth by reducing EphA2 expression in gastric cancer [11]. In agreement with their data, we have demonstrated that miR-520d-3p level was conspicuously elevated in melanoma tissues and cells. Inhibition of miR-520d-3p boosted cell growth and repressed apoptosis in melanoma cells. Moreover, miR-520d-3p suppressed the progression of melanoma cells by inhibiting *ASF1B* expression.

In our study, we have clarified that miR-520d-3p hampered the development of melanoma by inhibiting *ASF1B* expression, which could be a promising target for the clinical treatment of melanoma. Additionally, we will study the correlation between the miR-520d-3p-*ASF1B* axis and the prognosis of patients with melanoma and cancer histopathology once the opportunity arises. Furthermore, although we have confirmed the interaction between miR-520d-3p and *ASF1B* in melanoma cells in our study, the signaling pathways involved need to be elucidated. Moreover, the biological role of miR-520d-3p-*ASF1B* axis in melanoma needs to be verified and determined using animal models.

## Conclusions

In conclusion, through our study, we clarified for the first time that miR-520d-3p hampered the development of melanoma by inhibiting *ASF1B* expression, which could be a promising target for the clinical treatment of melanoma. Additionally, future studies on melanoma screening and diagnosis may be carried out based on the regulatory mechanism of the miR-520d-3p-*ASF1B* axis.

## Highlights

1. *ASF1B* was upregulated in melanoma cells and facilitated proliferation and adhesion, but repressed cell apoptosis of melanoma cells.

2. miR-520d-3p targeted *ASF1B*.

3. miR-520d-3p was downregulated in melanoma cells and restrained proliferation and adhesion, but repressed cell apoptosis of melanoma cells.

4. miR-520d-3p hampered melanoma cell progression by inhibiting *ASF1B*.

## Data sharing statement

The datasets used and analyzed during the current study are available from the corresponding author on reasonable request.
